# The ultrasound competency assessment tool for four-view cardiac POCUS

**DOI:** 10.1186/s13089-021-00237-3

**Published:** 2021-09-27

**Authors:** Colin Bell, Natalie Wagner, Andrew Hall, Joseph Newbigging, Louise Rang, Conor McKaigney

**Affiliations:** 1 Department of Emergency Medicine & Cumming School of Medicine, Emergency Physician, Calgary Zone, Alberta Health Services, University of Calgary, 7007 14th St. SW, Calgary, AB T2V1P9 Canada; 2 Department of Emergency Medicine, Queen’s University, 76 Stuart St Kingston, Kingston, ON K7L2V7 Canada; 3 Department of Biomedical & Molecular Sciences, Queen’s University, 74 Union St Kingston, Kingston, ON K7L3N6 Canada; 4 Office of Professional Development & Educational Scholarship, Queen’s University, 68 Barrie St Kingston, Kingston, ON K7L3N6 Canada; 5 Royal College of Physicians and Surgeons Canada, 774 Echo Drive, Ottawa, ON K1S5N8 Canada; 6 Department of Emergency Medicine, The Ottawa Hospital and University of Ottawa, 1053 Carling Ave, E-Main, Room EM-206, Box 227, Ottawa, ON K1Y4E9 Canada

**Keywords:** POCUS, Medical education, Cardiac ultrasound, Assessment, Competency-based medical education, Focused echocardiography

## Abstract

**Background:**

Point-of-care ultrasound (POCUS) has been recognized as an essential skill across medicine. However, a lack of reliable and streamlined POCUS assessment tools with demonstrated validity remains a significant barrier to widespread clinical integration. The ultrasound competency assessment tool (UCAT) was derived to be a simple, entrustment-based competency assessment tool applicable to multiple POCUS applications. When used to assess a FAST, the UCAT demonstrated high internal consistency and moderate-to-excellent inter-rater reliability. The objective of this study was to validate the UCAT for assessment of a four-view transthoracic cardiac POCUS.

**Results:**

Twenty-two trainees performed a four-view transthoracic cardiac POCUS in a simulated environment while being assessed by two observers. When used to assess a four-view cardiac POCUS the UCAT retained its high internal consistency ($\alpha =0.90)$ and moderate-to-excellent inter-rater reliability (ICCs = 0.61–0.91; *p*’s ≤ 0.01) across all domains. The regression analysis suggestion that level of training, previous number of focused cardiac ultrasound, previous number of total scans, self-rated entrustment, and intent to pursue certification statistically significantly predicted UCAT entrustment scores [F (5,16) = 4.06, *p* = 0.01; R^2^ = 0.56].

**Conclusion:**

This study confirms the UCAT is a valid assessment tool for four-view transthoracic cardiac POCUS. The findings from this work and previous studies on the UCAT demonstrate the utility and flexibility of the UCAT tool across multiple POCUS applications and present a promising way forward for POCUS competency assessment.

**Supplementary Information:**

The online version contains supplementary material available at 10.1186/s13089-021-00237-3.

## Background

Point-of-care ultrasound (POCUS) has become an integral diagnostic and interventional tool within medicine [1]. For physicians from many specialty backgrounds, POCUS skills are recognized as vital to their practice of medicine. Furthermore, multiple medical societies have made strong recommendations about the inclusion of POCUS applications into bedside patient care, such as incorporating focused cardiac ultrasound into the initial assessment of patients with cardiopulmonary instability and undifferentiated shock [2]. While many trainees and faculty engage in POCUS training, and some POCUS assessment tools already exist [3–8], a lack of reliable and streamlined competency assessment processes with demonstrated validity remains a significant barrier to widespread clinical integration [9].

The Ultrasound Competency Assessment Tool (UCAT) [10], which utilizes a modern global entrustment scale [11] has recently been presented as an alternative to traditional checklist-based scales. The UCAT was expert derived using Delphi methodology to be a rapid and easy-to-use assessment tool relevant across most POCUS applications. While the initial UCAT study provided some validity evidence for its use in the evaluation of the focused assessment with sonography for trauma (FAST), demonstrating strong inter-rater reliability and internal consistency, the UCAT was unable to discriminate between levels of training [10]. As the literature suggests that the FAST is a fairly simple POCUS assessment [12, 13], due to its large, accessible sonographic windows, it raises questions as to whether the lack of discriminatory ability was a result of the UCAT tool or the POCUS task itself. This limitation in the initial UCAT derivation study highlighted the need to explore the use of the UCAT with other POCUS applications [10].

A transthoracic cardiac POCUS, consisting of the cardinal four views (parasternal long, parasternal short, subxiphoid and apical four chamber), has a steeper learning curve than FAST and some other POCUS applications [12, 13]. Components of the four-view cardiac POCUS are regarded as more challenging due to smaller sonographic windows caused by intercostal spaces, variable location of the ideal interspace for cardiac visualization [14] and lung artifact. Occasionally changes in patient positioning and maneuvers coordinating image acquisition with the respiratory cycle are required. These factors can make image generation and image optimization more complex compared to other POCUS assessments such as the FAST. Thus, the objective of this study was to build on previous work and utilize the entrustment-based UCAT to assess POCUS competency for trainees performing a focused four-view cardiac ultrasound.

## Methods

### Study context

This study took place within the University of Calgary Emergency Medicine (EM) program. This program has two training streams with 20 trainees in the Royal College of Physicians and Surgeons specialist stream and 8 trainees in the Canadian College of Family Physicians enhanced skills Emergency Medicine stream. All trainees work in the same clinical environment and participate in shared academic days. In August 2019, trainees from both programs were invited to participate in this study during an existing academic day for oral exam practice. Informed consent was obtained from all trainees and participation in the study was voluntary. Performance had no impact on program academic standing. Ethics approval was provided by the University of Calgary (No. 18-0373) and Queen’s University Health Science Research Ethics Board (No. 6023366).

### Design

Prior to participating in the POCUS assessment, trainees completed a brief digital questionnaire on the Qualtrics survey platform (Provo, UT) quantifying their past ultrasound experience on different types of POCUS, including self-estimated previous number and types of POCUS scans performed, self-rated UCAT entrustment at POCUS (including all POCUS, and specifically FAST and four-view focused cardiac POCUS), intent to pursue ultrasound certification as a proxy for POCUS motivation, and previous exposure to ultrasound training.

Trainees were then assessed sequentially performing focused cardiac POCUS on a healthy, live simulated patient with suitable sonographic windows verified by one of the assessors (CM). Participants were first read a brief standardized stem by one of the assessors (CM), and then allowed 10 min to complete the station. The stem requested the examination of a previously healthy young male patient presenting with normal vital signs presenting and sudden onset acute dyspnea. The stem specifically requested the trainee perform and interpret a focused cardiac ultrasound consisting of the cardinal four two-dimensional (2D) views (parasternal long, parasternal short, subxiphoid and apical four chamber) and then articulate the subsequent investigations and management plan based on the POCUS results in the context of the stem and simulated patient. To standardize the assessment as much as possible, after completion of the scenario by each participant, the entire station including the POCUS machine placement and settings, bed position, and model positioning and draping were returned to their initial state. This was performed to blind the incoming trainee from actions performed by the previous participant. The focused cardiac ultrasound was selected because it has been demonstrated to be a more difficult POCUS application [12].

Two fellowship-trained sonographers watched each performance live (CB and CM), and independently assessed the trainees’ POCUS competency using the UCAT. The UCAT was designed to be user-friendly and applicable to a broad range of POCUS applications. The UCAT consists of four domains (preparation, image acquisition, image optimization, and clinical integration), each with behavioral descriptors, as well as a single overall entrustment score (Additional file 1). A detailed description of the UCAT development and initial implementation can be found elsewhere [10]. Of the two raters, one assessor (CB) was from a different training site and had not previously interacted with the residents in a clinical or teaching environment making him blind to participants’ previous POCUS experience.

### Statistical analysis

Descriptive statistics were calculated on the questionnaire responses and UCAT scores for the focused cardiac ultrasound. A paired t-test compared participants self-rated entrustment on FAST versus cardiac POCUS. Two-way random, Intra-class Correlation Coefficients (ICC) were used to evaluate the inter-rater reliability across each performance domain. Cronbach’s alpha was calculated to measure internal consistency, or agreement across performance domains. Raters’ scores were then averaged for each domain and combined to create a composite score across all domains for each participant. A standard multiple regression analysis was used to explore the impact of postgraduate year (PGY), previous number of POCUS scans (total), previous number of focused cardiac ultrasound scans, self-reported entrustment on an advanced cardiac POCUS, and intent to pursue a POCUS certification on UCAT scores. The regression was run twice, once using the composite score as the UCAT performance outcome and once using the entrustment score alone, to evaluate whether the two indices provided similar information. All statistical analyses were conducted with SPSS Version 25 (IBM SPSS Statistics, Armonk, New York, USA). Statistical significance was considered at $p\le 0.05$.

## Results

A total of 22/28 (79%) of the Calgary EM trainees participated in our study. Participants represented PGY1 to PGY4 only, with PGY5 trainees unavailable due to their rotation and study schedules. Participants had variable experience doing FAST and focused cardiac ultrasound (Table 1). The paired t-test suggested that participants’ self-rated entrustment for FAST was significantly higher than their self-rated entrustment for cardiac POCUS [t (21) = 6.5, *p* < 0.001). Participant self-ratings are also presented in Table 1.

**Table 1:** Previous POCUS experience and self-rated entrustment

PGY	n	Previous number of scans: FAST		Previous number of scans: four-view cardiac		Previous number of scans: total		Self-rated entrustment (1 to 5)	
		Average (SD)	Range	Average (SD)	Range	Average (SD)	Range	FAST	Cardiac POCUS
PGY1	2/4	37 (5)	31–41	7 (7)	0–15	112 (11)	99–120	3.0 (0)	1.67 (0.6)
PGY2	4/4	82 (41)	57–144	58 (42)	2–101	355 (146)	234–560	3.75 (0.5)	2.0 (1.4)
PGY3	4/4	118 (39)	70–150	37 (19)	15–55	408 (90)	320–496	4.75 (0.5)	3.5 (1.3)
PGY4	3/4	171 (112)	97–300	35 (14)	23–50	572 (371)	348–1000	4.67 (0.6)	3.33 (0.6)
PGY5	0/4								
CCFP-EM	8/8	54 (52)	0–150	12 (14)	0–34	186 (173)	3–477	3.75 (1.4)	2.37 (1.1)

Results of the intra-class correlation analyses suggested moderate-to-excellent inter-rater reliability across the UCAT domains, with ICC values of 0.61–0.91; *p*s ≤ 0.01 (Table 2; ICC guidelines from Koo and Li 2016) [8]. In all cases when the raters varied, the variation was only by one point on the five-point scale. The results of the Cronbach’s alpha suggested high internal consistency among performance categories in the UCAT ($\alpha =0.90).$

**Table 2:** Mean scores across raters and Intra-class Correlations Coefficients per domain

UCAT domain	Rater 1 (CB) Mean (SD)	Rater 1 (CB) Range	Rater 2 (CM) Mean (SD)	Rater 2 (CM) Range	Average of Two Raters	ICC (Absolute Agreement)	*p*-value
Preparation	2.45 (0.51)	2.0–3.0	2.60 (0.67)	1.0–3.0	2.52	0.71	0.003*
Image acquisition	1.64 (0.66)	1.0–3.0	1.77 (0.68)	1.0–3.0	1.70	0.86	< 0.001*
Image optimization	1.64 (0.73)	1.0–3.0	1.91 (0.75)	1.0–3.0	1.78	0.61	0.01*
Clinical integration	2.14 (0.56)	1.0–3.0	2.32 (0.65)	1.0–3.0	2.23	0.68	0.005*
Entrustment	2.55 (1.14)	1.0–5.0	2.76 (1.09)	1.0–5.0	2.65	0.91	< 0.001*

*Significance considered at *p* ≤ 0.05

**Fig. 1:**
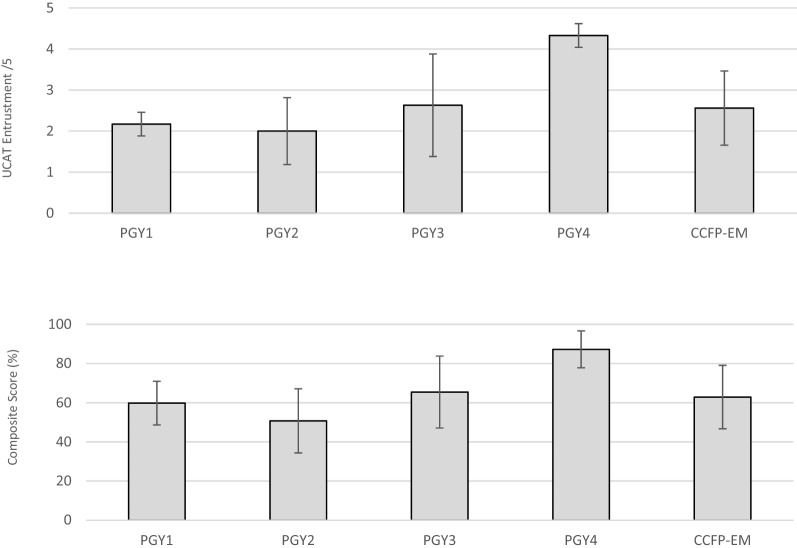
UCAT score by postgraduate year (PGY)

Lastly, the multiple regression analysis suggested that PGY, previous number of focused cardiac ultrasound, previous number of total scans, self-rated entrustment, and intent to pursue certification statistically significantly predicted UCAT entrustment scores [F (5,16) = 4.06, *p* = 0.01; R^2^ = 0.56]. While no variable was a statistically significant predictor on its own (Table 3), the factor that accounted for the most variance was self-rated entrustment on POCUS. When the analysis was run with UCAT composite scores as the outcome, the results were very similar (Table 3). Composite UCAT scores and entrustment scores alone are displayed by PGY in Fig. 1. Notably, while PGY was not a significant factor in the regression analysis, descriptive statistics suggest that the PGY4s UCAT scores were the highest among study participants, and that there was larger variation around the mean among PGY3s, compared to other cohorts (Fig. 1).

**Table 3:** Predictive factors for UCAT score

Factors	Entrustment scores beta coefficient	Entrustment scores t value	Entrustment scores *p-*values (sig.)	Composite scores beta coefficient	Composite scores t value	Composite scores *p-*values (sig.)
Number of total scans	0.43	1.46	0.16	0.32	1.02	0.32
PGY	− 0.11	− 0.40	0.70	− 1.08	− 0.36	0.73
Number of previous focused cardiac ultrasound scans	− 0.28	− 1.24	0.23	− 0.18	− 0.76	0.46
Intent to pursue POCUS certification	− 0.31	− 1.08	0.30	− 0.18	− 0.60	0.56
Self-rated entrustment focused cardiac ultrasound	0.47	1.97	0.07	0.54	2.15	0.05
All factors combined	R^2^	F value (df)	*p*-value (sig)	R^2^	F value (df)	*p*-value (sig)
	0.56	F (5,16) = 4.06	0.01	0.50	F (5,16) = 3.21	0.03

## Discussion

The UCAT was created to be a universally applicable, entrustment-based, POCUS competency assessment tool [10]. An early argument for validity of the UCAT for the assessment of FAST competency has been previously published as part of its derivation [10]. This current study sought to expand on previous work and explore the use of the UCAT with a more technically difficult application, the four-view cardiac POCUS. Using Messick’s validity framework [15], we articulate how this study provides further validity evidence for the UCAT in the domains of internal structure validity (internal consistency, inter-rater reliability) and relations with other variables. We then propose how individuals and programs engaging in POCUS training and assessment can utilize the UCAT in their programs of assessment.

### Internal structure validity

Our results suggest that the UCAT maintains high internal consistency ($\alpha =0.90)$ when used to assess cardiac POCUS, and that all four domains (preparation, image acquisition, image optimization, clinical integration) as well as the entrustment rating, captured relevant, but not redundant, information on POCUS competency. As per Koo and Li’s (2016) Intra-Class Correlation (ICC) guidelines [16], the results of this study also demonstrate that the UCAT domains have moderate-to-excellent inter-rater reliability (ICCs = 0.61–0.91). This level of agreement was achieved with one local rater who was aware of trainees’ level and general ability, and one external rater who was blinded to the trainee’s experience, which speaks to the strength of the tool. The inter-rater reliability and internal consistency findings are consistent with the UCAT results found when assessing a FAST [10], suggesting that the UCAT is a reliable POCUS assessment tool across multiple POCUS applications.

Consistent with previous literature [17, 18], the entrustment category had the highest Intra-class Correlation Coefficients (ICC = 0.91). Global assessment of observable competencies using entrustment scales has become commonplace in postgraduate education, where the typical goal is to capture the assessors tacit capacity for judgement of the need for supervision of a trainee [19]. The UCAT both facilitates this global assessment of competency via an overall entrustment score and also provides individual domain scoring which may serve to facilitate feedback and coaching for improvement (or assessment *for* learning) [20]. The use of domain specific items and overall entrustment aligns with modern programs of assessment in North America, including the structure of entrustable professional activities and milestones in the Canadian Competency by Design framework [21], and milestones and sub-competencies in the ACGME Milestones Project [22]. Additionally, as our work identified similar validity evidence for the domain specific scores and the overall entrustment score, we believe both are critical and reliable features of the UCAT.

### Correlations with other variables

The argument for the validity of the UCAT is further bolstered by its correlation with other variables that are traditionally used as indicators of POCUS competency, such as PGY, previous experience, intent to pursue POCUS certification, and self-rated entrustment or confidence. The results of this study suggest that together, these variables predicted UCAT scores [F (5,16) = 4.06, *p* = 0.01; R^2^ = 0.56]. Although no variables were statistically significant predictors on their own, there were notable differences in mean UCAT scores based on PGY. Despite the PGY2-4 s having similar experience with POCUS scans (Table 1), the results of this study suggest that the PGY4 cohort outperformed the other trainees on the cardiac POCUS exam with a median entrustment score of ≥ 4 *out of* 5 on the UCAT. These results align with previous literature suggesting the number of previous scans and PGY year are not sufficiently reliable indicators of competency in isolation [12, 23–25], reinforcing the need for an assessment tool like the UCAT.

Moreover, in typical competency-based medical education frameworks, it is becoming widely recognized that a score of 4 (e.g., I needed to be there just in case) or above is often considered an acceptable indicator of competency for trainees. In this study, only the PGY4s had an average UCAT performance at 4 or higher. This is in sharp contrast to the initial FAST UCAT where all trainees who had previously completed the Canadian Association of Emergency Physicians POCUS curriculum [26] achieved a level of 4 or higher. This difference in entrustment scores between FAST and four-view cardiac assessments speaks to the discriminatory capacity of the tool. First, it aligns with the general relationship between expected trajectories of competency in FAST versus four-view cardiac. The difference in UCAT scores for FAST and four-view cardiac POCUS also aligns with prior research which suggests trainees are able to rapidly achieve competency with the FAST [12] and four-view cardiac POCUS requires more practice and training before competency is achieved [12, 27]. The results of the pre-session questionnaire in this study also support this, as trainees reported a high degree of FAST experience and self-rated entrustment, and a lower self-rated entrustment and experience with four-view cardiac POCUS. Taken together, these findings reinforce that a four-view cardiac POCUS is more difficult than FAST, and that the UCAT is able to capture differences in task difficulty.

### UCAT implementation moving forward

POCUS is a core competency for graduating EM residents [22, 28], and practicing physicians. At present there is no consensus on which assessment tools are best to measure POCUS competency [3, 29]. This study shows that the UCAT can be used to effectively assess POCUS competency in a consistent manner, and, taken with previous work, that it is accurate across more than one POCUS study type. The UCAT's high inter-rater reliability and internal consistency means that it can be used as a direct observation tool for both low- and high-stakes assessments. Broad usage of the simple and adaptable UCAT would standardize the assessment of POCUS competency across EM and other specialties. This may simplify credentialling across institutions and professional associations.

### Limitations

There are a number of limitations that must be mentioned with respect to the current study. This study occurred at a single center and was used with a small population of trainees who had largely undergone core ultrasound training emphasizing the subxiphoid cardiac view [26]. There were very few learners who had not had some exposure to the four-view cardiac POCUS, although most had only completed less than 30 studies. While this may be a limited exposure for this POCUS type, it is aligned with the findings on trainee experience from the previous UCAT study [10]. While all available trainees participated in the study, a post hoc power analysis suggested this study was underpowered (0.40), limiting our ability to identify individual predictors [30]. Moreover, the factors evaluated in the regression were treated as independent factors, and while there is some variability within and between PGY, number of studies completed, and entrustment, it is difficult to truly disentangle these measures. All background digital survey data were self-reported. Due to the charting and billing structure in this residency program, there was no way of verifying the quantity of POCUS studies previously performed by each resident. Both raters in this study were involved in the derivation and the FAST validation of the UCAT, thus further work is needed to confirm inter-rater reliability with assessors less familiar with the tool. Lastly, at present, assessments of the UCAT have taken place in a simulated environment. As a result, the documentation and comprehension components of the UCAT were assessed through the trainee’s verbal articulation of their subsequent plan based on the findings from their POCUS study in the context of the stem rather than in a written medical record. Previously derived similar tools such as the Resuscitation Assessment Tool have shown a smooth transition from the simulated to the clinical environment [31], the UCAT should be trialed and evaluated in the clinical environment to ensure that it remains an effective evaluation tool for the assessment of POCUS competency.

## Conclusions

The UCAT was created to be a universally applicable, simple, entrustment-based, POCUS competency assessment tool [10]. Previous work has demonstrated strong validity evidence in the use of the UCAT for assessing FAST [10]. This study has expanded on this work, replicating and confirming the UCAT’s validity evidence with respect to inter-rater reliability, internal consistency, and comparison with other variables such as PGY, previous number of four-view cardiac POCUS, previous number of total scans, self-rated entrustment, and intent to pursue certification together. Further, this study has expanded the evidence for its discriminatory capacity through a demonstration of its performance characteristics in the more difficult four-view cardiac POCUS examination when compared to FAST. Taken together, the findings from this work and previous studies on the UCAT demonstrate the utility and flexibility of the UCAT tool across multiple POCUS applications and present a promising way forward for measuring trainee POCUS competency.

## Supplementary Information

**Additional file 1:** The Ultrasound Comptency Assessment Tool.

## Data Availability

All data are stored at the Queen’s University Department of Emergency Medicine Research Office. The digital data are housed in the encrypted database at Queen’s University. Deidentified data are available from the corresponding author on reasonable request.

## Abbreviations

POCUS: Point-of-care ultrasound
UCAT: Ultrasound competency assessment tool
FAST: Focused assessment with sonography in trauma
