# Supplementary figures and images for: The ultrasound competency assessment tool for four-view cardiac POCUS
Source: Ultrasound J. 2021 Sep 27;13:42. doi: 10.1186/s13089-021-00237-3 (PMC8476706; doi:10.1186/s13089-021-00237-3)

Supplemental Materials 1 – The Ultrasound Comptency Assessment Tool


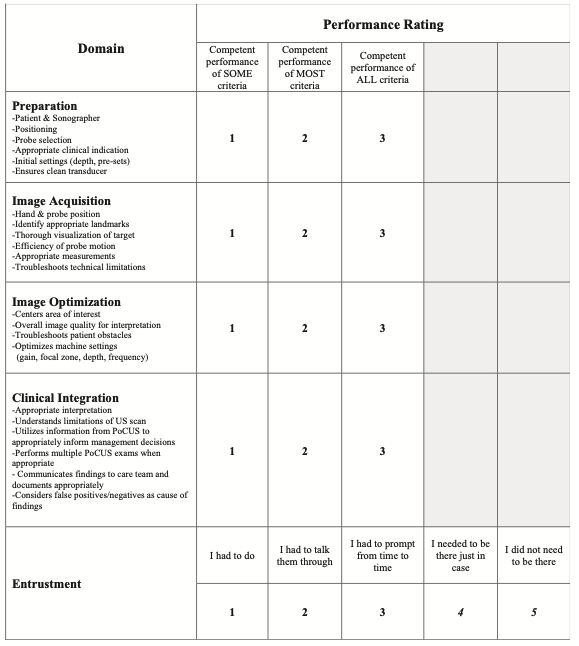

Supplement: Supplementary file 1 — Additional file 1: The Ultrasound Comptency Assessment Tool. [file 13089_2021_237_MOESM1_ESM.docx]
